# Comparison of central laboratory HbA1c measurements obtained from a capillary collection versus a standard venous whole blood collection in the GRADE and EDIC studies

**DOI:** 10.1371/journal.pone.0257154

**Published:** 2021-11-15

**Authors:** David M. Nathan, Heidi Krause-Steinrauf, Barbara H. Braffett, Valerie L. Arends, Naji Younes, Paula McGee, Claire Lund, Mary Johnson, Gayle Lorenzi, Xiaoyu Gao, Michael W. Steffes, John M. Lachin, the GRADE Research Group, the DCCT/EDIC Research Group

**Affiliations:** 1 Massachusetts General Hospital, Harvard Medical School, Boston, MA, United States of America; 2 The Biostatistics Center, Milken Institute School of Public Health, The George Washington University, Rockville, MD, United States of America; 3 Advanced Research and Diagnostic Laboratory, Department of Laboratory Medicine and Pathology, University of Minnesota, Minneapolis, MN, United States of America; 4 International Diabetes Center, Health Partners Institute, Minneapolis, MN, United States of America; 5 University of California, San Diego, La Jolla, CA, United States of America; Medizinische Universitat Graz, AUSTRIA

## Abstract

**Background:**

We compared HbA1c values obtained from capillary blood collection kits versus venous whole blood collections in study participants with type 1 or type 2 diabetes.

**Methods:**

A total of 122 subjects, 64 with type 2 diabetes participating in the Glycemia Reduction Approaches in Diabetes: A Comparative Effectiveness (GRADE) Study and 58 with type 1 diabetes from the Epidemiology of Diabetes Interventions and Complications (EDIC) Study, participated in the validation study. Capillary tubes were filled by fingerstick by the participants on the same day as the collection of venous whole blood samples in EDTA-containing test tubes and were mailed to the central laboratory. HbA1c in all samples was measured with the same high-performance liquid chromatography. GRADE participants also completed a questionnaire on the ease of performing capillary collections.

**Results:**

Participants from 22 clinical centers (GRADE n = 5, EDIC n = 17) were between 35 and 86 years of age, with 52% male and diverse race/ethnicities. Venous HbA1c results ranged between 5.4–11.9% (35.5–106.6 mmol/mol) with corresponding capillary results ranging between 4.2–11.9% (22.4–106.6 mmol/mol). The venous and capillary results were highly correlated (R^2^ = 0.993) and 96.7% differed by ≤0.2% (2.2 mmol/mol). Of participants surveyed, 69% indicated that the instructions and collection were easy to follow and 97% felt the collection method would be easy to do at home.

**Conclusions:**

The capillary blood HbA1c results compared well with the conventional venous whole blood results. The capillary kits can be employed in other studies to reduce interruption of critical data collection and potentially to augment clinical care when in-person visits are not possible.

## Introduction

Glycated hemoglobin measurements have played a dominant role in assessing long-term, mean glycemia in clinical research and clinical care for almost 40 years [1]. Based on the demonstrated importance of mean glycemia in the pathogenesis of long-term complications in type 1 and type 2 diabetes [2, 3]. HbA1c, usually measured every 3 to 6 months, remains the primary measure to assess and compare glycemia in clinical trials and clinical care. Numerous assays for HbA1c have been standardized by the National Glycohemoglobin Standardization Program, but all of them require use of venous whole blood stored at refrigerated temperatures and testing within 1–7 days to maintain stability and integrity [1]. We evaluated a kit which allowed participants to collect capillary samples by fingerstick and mail them to a central laboratory where they were assayed with the same high-performance liquid chromatography (HPLC) method as used on venous whole blood samples. The capillary kit was originally developed for the Glycemia Reduction Approaches in Diabetes: A Comparative Effectiveness (GRADE) study [4, 5] for continuity of assessment of HbA1c measured at quarterly visits when participants were unable to attend usual study visits owing to infirmity or other reasons. The Epidemiology of Diabetes Interventions and Complications (EDIC) study [6] completed a similar validation of capillary collections for its participants. After validation, the capillary kit was widely employed in the GRADE and EDIC studies when COVID-19 epidemic-related restrictions threatened to interrupt collection of HbA1c measurements. We report the validation results of the capillary kit for HbA1c data collection.

## Materials and methods

### Study design

The GRADE and EDIC overall study designs have been reported previously [4, 6]. Each study conducted a capillary collection kit validation study to evaluate the feasibility of the capillary collection process, the accuracy of HbA1c results obtained from participant self-collected capillary blood samples compared to venous whole blood samples collected on the same day during an in-clinic study visit, and the suitability of the kit and mailing process for collections taking place outside the clinic. Both studies utilized identical procedures for the collection and mailing of capillary and whole blood sample collections to the same central laboratory for analysis. The validation study protocol, informed consent and procedures were initially developed by the GRADE Study and adapted by the EDIC Study. Each clinical site that participated in the validation study obtained local institutional review board approval. The George Washington University Biostatistics Center served as the Coordinating Center for both studies and the Advanced Research and Diagnostic Laboratory (ARDL) at the University of Minnesota served as the Central Biochemistry Laboratory for both studies.

### Participants

The participant baseline characteristics for GRADE and EDIC have been previously reported [5, 6]. GRADE and EDIC studied participants with type 2 and type 1 diabetes, respectively. Of the 36 GRADE clinical centers, 5 volunteered to participate and recruited participant-volunteers for the validation study. The GRADE capillary kit validation study was conducted between March 2015 and August 2016. GRADE clinical centers were recruited to represent wide-spread geographic locations across the US, with varying climatologic conditions, and willingness to conduct the validation study. Participants were eligible for the study if they had an upcoming visit in which an HbA1c was measured. Likewise, in EDIC, 17 of the 27 clinical centers volunteered and recruited participant-volunteers between April and June, 2016. In EDIC, 4 clinics chose not to participate based on IRB obstacles and 6 clinics chose not to participate because either the participants that would be seen for an annual visit during the time frame specified for the study did not meet the HbA1c criteria or the clinic had other reasons for not participating.

In both studies, potential participants were identified based on previous HbA1c levels, aiming to enrich the number of samples with HbA1c greater than 7.5% (58.5 mmol/mol) to ensure sample validation across the spectrum of HbA1c results. All participants gave written informed consent.

### Procedures

The capillary and whole blood collections were obtained during non-fasting study visits at 6 months post-randomization or later in GRADE, and at regularly scheduled annual study visits in EDIC. Participants were consented and given the capillary collection kit along with detailed written instructions with photographs that described the contents of the kit with step-by-step collection and mailing instructions (S2 Fig). Participants performed the capillary blood collection by fingerstick in the clinic and packaged the sample for mailing. Clinic study staff were available to answer questions and clarify instructions with the goal of facilitating the sample collection process and addressing issues that might arise when the collection was performed independently at home. The participant-packaged capillary sample was mailed by study staff to the central laboratory via US mail at ambient temperature on the day of collection using a pre-addressed and stamped mailer. On the same day, the study staff collected the EDTA-anticoagulated venous blood as specified in the GRADE and EDIC Study protocols and shipped the sample to the central laboratory per usual study procedures. The GRADE Study participants completed a questionnaire after the collection to assess the practical aspects of performing the capillary collection and feasibility of completing the capillary collection at home.

### Laboratory methods and kit description

All blood samples were analyzed at the Advanced Research and Diagnostic Laboratory at the University of Minnesota. The whole blood and capillary samples were assayed on the Tosoh HPLC Glycohemoglobin Analyzer (Tosoh Medics, Inc., San Francisco CA 94080), an automated high performance liquid chromatography method. The assay is calibrated monthly using standard samples supplied by the National Glycohemoglobin Standardization Program (NGSP) and twice every year with samples provided through a program of the International Federation of Clinical Chemistry and Laboratory Medicine (IFCC). The method’s coefficient of variation is 1.2% at a mean HbA1c of 5.34% (34.9 mmol/mol) and 0.55% at a mean HbA1c of 10.11% (87 mmol/mol).

The capillary collection kits for each study were designed by the Advanced Research and Diagnostic Laboratory at the University of Minnesota and used the BioRad Hemoglobin Capillary Collection System (HCCS) [7]. The HCCS includes a sample preparation vial containing a solution of EDTA and potassium cyanide, a 5 μL plastic capillary tube, and a tube holder for positioning the capillary tube during collection of the fingerstick sample. In addition to the HCCS supplies, all other supplies required to collect the blood sample were included in the kit, including lancets, alcohol wipes and gauze. Supplies required to ship the capillary blood according to US Postal Service (USPS) regulations were provided in the kit, including an appropriately-labeled, postage paid mailing box, a biohazard-marked bag and absorbent material to capture any sample leakage.

### Statistical analysis

The study did not include duplicate measurements of individual specimens to assess the precision or reproducibility (statistically, the reliability) of either assay. However, precision was assessed indirectly through its relationship to the strength of the correlation of the two methods since the maximum possible expected squared correlation is *E*(*R*^2^) = *ρ_x_ρ_y_* where *ρ_x_* and *ρ_y_* are the reliability coefficients of the two variables [8]. For a given variable, *ρ* is the proportion of total variation that is due to differences in the true values, or 1–*ρ* is the proportion of total variation among values that is due to various sources of error. These coefficients are what are usually evaluated in a split-duplicate analysis. Thus, the reliability of either assay is no less than the value of the R^2^.

An equation to convert a capillary HbA1c to a corresponding venous whole blood HbA1c was obtained through least squares regression by regressing venous HbA1c on capillary HbA1c. The regression equations and their R^2^ separately for GRADE, EDIC, and both studies combined are reported. Bland-Altman plots of the difference between capillary and venous HbA1c against their average are presented for each study separately as well as for both studies combined. A lowess smoother is added to the plots to show possible trends. All analyses were done using R 4.0.1 or SAS version 9.4 (Copyright SAS Institute Inc.).

## Results

Collection from GRADE participants commenced in March 2015 and was completed in August 2016. A total of 70 subjects from 5 clinical centers participated (10–16 from each center). Of these, 64 participants had measurable HbA1c results for both the venous sample and corresponding capillary blood sample. The 6 GRADE subjects not included did not have capillary results reported due to shipping delays resulting in late arrival of four samples (13, 14, 16, and 22 days after samples obtained), and improper collection of capillary samples with too little blood volume in two samples. In EDIC, the capillary kit validation study was performed between April and June 2016 at 17 of the 27 EDIC clinical centers with 1–5 participants at each center, for a total of 58 participants. The geographic centers from both studies included 21 clinical centers in the US and 1 in Canada, with a wide distribution across North America (S1 Fig). The average delivery time via the USPS from sampling to arrival in the Minnesota laboratory was 6 (range 1–22) days.

On average, the GRADE participants (n = 64) were 56 (range 35–86) years old, 39% women and 50% self-identified as White, 17% American Indian/Alaskan Native, 17% Asian/Native Hawaiian or Pacific Islander, 9% African-American and 5% Hispanic. The EDIC participants (n = 58) had an average age of 57 (range 44–70) years with 57% women and 97% self-identified as White. One EDIC participant had a venous HbA1c of 8.1% (65 mmol/mol) but a capillary HbA1c of 4.2% (22.4 mmol/mol) that was confirmed on repeat testing. Thus, this discrepancy appears not to be attributable to sources of error (variation) within the central laboratory, but rather attributable to sources of variation outside of the laboratory (shipping, handling, labeling, etc.). Accordingly, this extreme outlier was excluded from the regression and Bland-Altman figures, but otherwise included.

**Fig 1:**
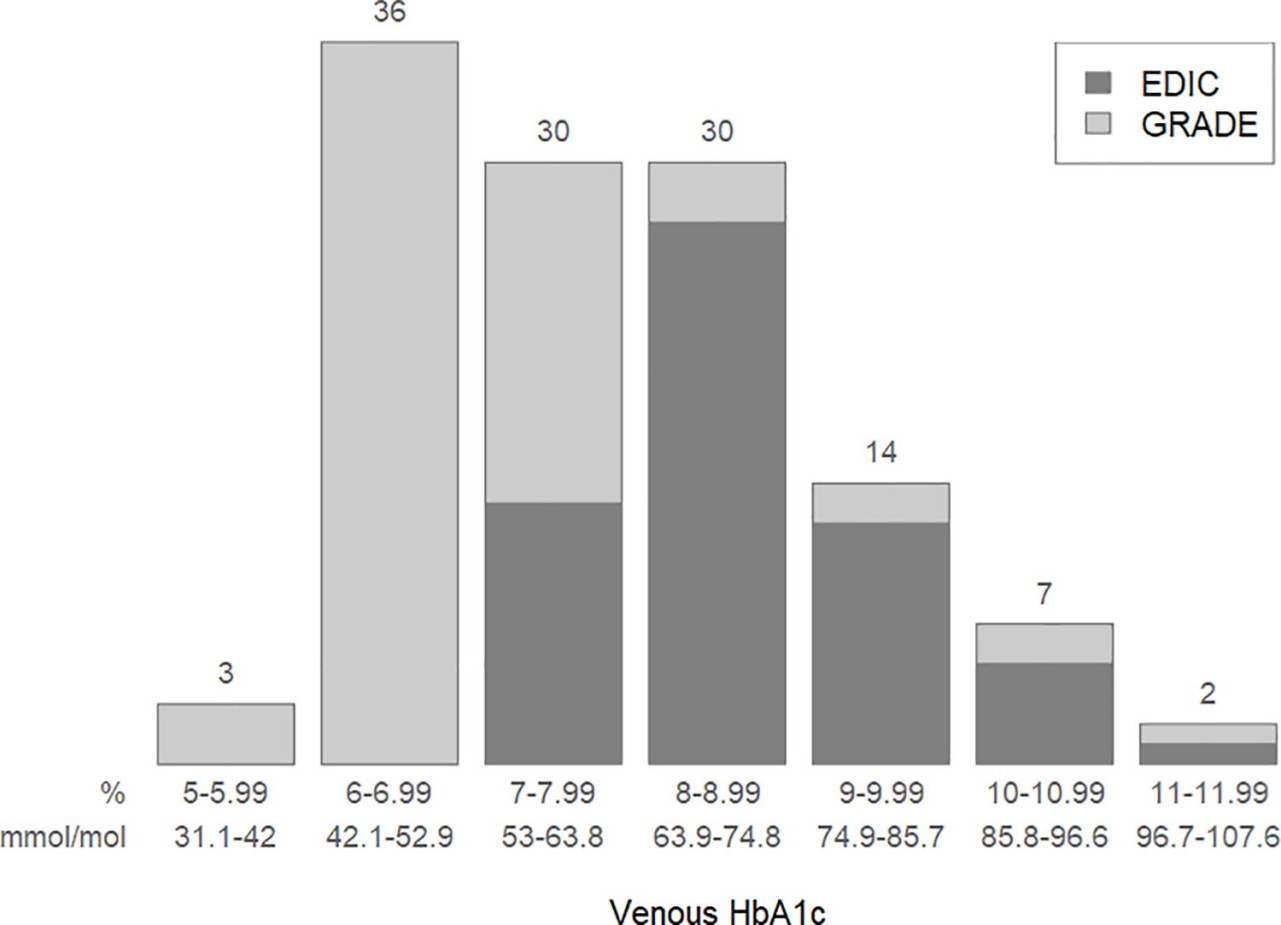
Distribution of HbA1c values from venous samples.

Table 1 presents the range for the venous and corresponding capillary HbA1c results for the GRADE and EDIC participants. Among GRADE participants, venous HbA1c results ranged between 5.4–11.9% (35.5–106.6 mmol/mol) with corresponding capillary results ranging from 5.3–11.9% (34.4–106.6 mmol/mol). Among the EDIC participants, the range of venous HbA1c results was 7.2–11.2% (55.2–98.9 mmol/mol) with corresponding capillary HbA1c results from 4.2–11.0% (22.4–96.7 mmol/mol), including the one outlier value. The distribution of the venous HbA1c values for each study and overall for both studies is presented in Fig 1.

**Table 1:** Venous and capillary HbA1c results.

		GRADE (n = 64 type 2 diabetes)		EDIC (n = 58 type 1 diabetes)	
		Venous HbA1c	Capillary HbA1c	Venous HbA1c	Capillary HbA1c
Mean (SD)	%	7.11 + 1.16	7.09 + 1.18	8.71 ± 0.92	8.62 ± 1.09
	mmol/mol	54.2 + 12.7	54 + 12.9	71.7 ± 10.1	70.7 ± 11.9
Median (IQR)	%	6.8 (6.4, 7.4)	6.8 (6.4, 7.4)	8.5 (8.1, 9.3)	8.6 (8.0, 9.2)
	mmol/mol	50.3 (46.5,57.7)	50.8 (46.5,57.4)	69.4 (65,77.9)	70 (64.2,77.1)
Range	%	5.4–11.9%	5.3–11.9%	7.2–11.2%	4.2–11.0%
	mmol/mol	35.5–106.6	34.4–106.6	55.2–98.9	22.4–96.7

**Fig 2:**
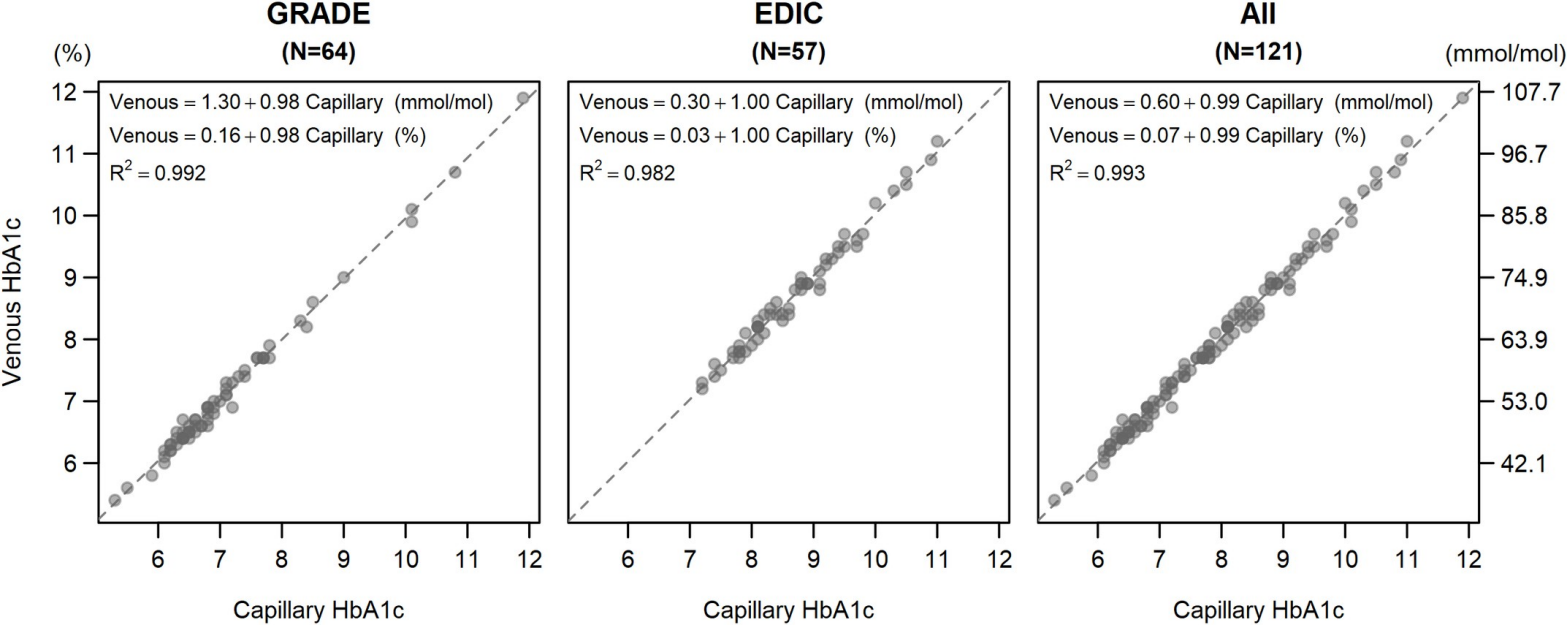
Regression plots of capillary and venous HbA1cs.

**Fig 3:**
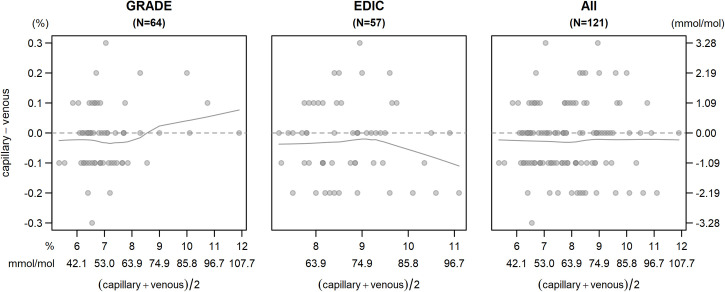
Bland-Altman plots.

The capillary and venous measurements and a simple regression equation with no covariate adjustments along with the regression lines for each study and the studies combined are shown in Fig 2. In the combined analysis, the regression relationship is Capillary HbA1c = 0.0671 + 0.9942 x Venous HbA1c which yields nearly perfect equality with an R^2^ of 0.993, indicating 99.3% of the variation in the capillary values is explained by the variation in the venous values. The Bland-Altman plots for each study and overall are in Fig 3. All but one of the discrepancies between capillary and venous samples were between -0.3 to 0.3 (-3.3 to 3.3 mmol/mol), with 98 (80.3%) between -0.1 and 0.1 (-1.1 to 1.1 mmol/mol) and all but four sample sets (96.7%) between -0.2 and 0.2 (-2.2 to 2.2 mmol/mol). The plots do not suggest any marked heteroscedasticity, and the lowess smoothers do not show a significant trend. In particular, there is no indication that the discrepancies increase markedly with higher HbA1c.

While the study did not include measurement of duplicate specimens to assess precision or reliability, a measure of reproducibility, the relationship between the R^2^ and reliabilities ensures that the reliability of either method is no less than the R^2^ value, or no less than 0.993 in our case [8]. This means that no more than 1–0.993 or 0.007 (less than 1%) of the variation in either assay is due to random sources of error.

All GRADE participants, most of whom did not routinely perform self-monitoring of blood glucose measurements, completed the participant use survey. Twenty (31%) of GRADE participants reported having one or more problems with the sample collection. These included issues with the instructions, fingerstick collection, use of the materials included in the kit and packaging instructions. Overall, 69% of GRADE participants reported that the instructions were easy to follow and 97% felt that the collection would be an easy-to-use option for home use if they were not able to attend clinic visits.

## Discussion

The GRADE and EDIC validation studies, individually and combined, demonstrated that the HbA1c results obtained from fingerstick capillary kits and mailed to a central laboratory were comparable and highly correlated with those obtained from routine venous samples. The results were consistent across the range of HbA1c measurements from 5% up to 12% (31.1–107.7 mmol/mol). The use of capillary specimens obtained by staff, not the patients, in a pediatric clinic setting was shown almost thirty years ago to provide results that correlated well with venous sample results in a single center [9]. The same investigators also demonstrated that mailed capillary samples, again obtained by staff, were stable with storage and compared well to other capillary samples that were mailed from and to a single center [10]. The current study adds important information, especially in the setting of restrictions on in-person visits imposed by COVID. Specifically, we have demonstrated that fingerstick sampling and filling of capillary tubes can be accomplished satisfactorily by study participants in multiple centers and that the results of mailed specimens from all over North America correlated highly with venous samples using current-day assays. A recently published study demonstrated similarly high correlations of HbA1c results from venous and capillary samples obtained at four centers and mailed to a central laboratory [11].

Participants in the validation study were representative of individuals with type 2 diabetes or type 1 diabetes enrolled in each study. With minimal training, study participants were able to implement use of the capillary kit collections. The overall feedback from those who completed the survey, most of whom did not routinely perform fingerstick self-monitoring, indicated that the instructions provided were sufficient and participants were positive regarding the ease of completing the collection and packaging the capillary sample for mailing. Based on the high correlation of results, both GRADE and EDIC subsequently implemented the capillary collections to augment remote or virtual visits when in-person evaluations were not possible.

The GRADE Study is following a cohort of more than 5000 and EDIC follows more than 1000 participants. Following the validation study, the GRADE Study developed a video for participants and a postcard to augment the written instructions (S3 Fig). Minor refinements were made to the kit and instructions based on the validation study results. In both studies, the capillary kits have been used for participants who for any reason were not be able to attend an in-person visit. More recently, many study visits were of necessity conducted remotely due to COVID restrictions and use of the capillary kits increased. Between the two studies, more than 6000 capillary kits were used between March 1, 2020 and April 30, 2021 to augment phone visits (~5800 collections in GRADE with results reported for 95%, ~400 collections in EDIC with results reported for 97%). Therefore, less than 5% of samples either did not arrive in a timely fashion, were insufficient for analyses, or were otherwise unsutable for measurement.

While this capillary tube-based approach has been successfully validated, there are some limitations in our study that may affect use in other studies. First, not all of the samples mailed by USPS arrived within the allowable timeframe to be analyzed. Similarly, a few capillary collections did not contain enough sample to be analyzed. Nevertheless, this only affected <5% of participant samples. Second, one participant had capillary and venous samples that exhibited a marked discrepancy in results. We have no explanation for the discrepancy, and it is not clear whether the capillary or venous sample was incorrect, although the very low capillary value suggests that it was problematic. However, consideration of HbA1c values in practice is usually made in the context of the patient’s history of treatment and past results and thus a major discrepancy, as with any aberrant result, should be apparent. In the clinical setting, the capillary tubes have not received FDA clearance and cannot currently be recommended

## Conclusion

The current validation studies support the use of participant-collected and shipped capillary blood samples for measuring HbA1c. The regression equation established herein allows conversion from capillary to venous results over a HbA1c range from 6 to 12% (42.1–107.7 mmol/mol). Of note, the differences between the raw data and the converted values are miniscule. With appropriate laboratory support, capillary kits can be used to ensure more complete HbA1c data collection in study participants who, for any reason, may not be able to attend in-person visits. Moreover, capillary HbA1c measurements may ameliorate large-scale interruptions in diabetes clinical research imposed by public health threats such as the COVID pandemic. Finally, such capillary kit assays may potentially be applied in the setting of increasing telemedicine as part of routine clinical care.

## Supporting information

S1 Table Capillary and venous sample data. (PDF)

S1 Fig Map of clinical centers. (PDF)

S2 Fig Participant instructions for capillary blood collection. (PDF)

S3 Fig Participant video instruction postcard for capillary blood collection. (PDF)

S4 Fig GRADE Research Group. (PDF)

S5 Fig DCCT/EDIC Research Group. (PDF)
