# Supplementary material for: Comparison of central laboratory HbA1c measurements obtained from a capillary collection versus a standard venous whole blood collection in the GRADE and EDIC studies
Source: PLoS One. 2021 Nov 15;16(11):e0257154. doi: 10.1371/journal.pone.0257154 (PMC8592405; doi:10.1371/journal.pone.0257154)
Supplement: S1 Table — (PDF) [file pone.0257154.s001.pdf]

S1 Table. Capillary and Venous Sample Data

March 29, 2021

Capillary HbA1c (%)	Venous HbA1c (%)	Study
8.4	8.6	EDIC
9.2	9.3	EDIC
8.1	8.2	EDIC
8.3	8.4	EDIC
9.1	8.8	EDIC
7.7	7.7	EDIC
7.8	7.8	EDIC
9.7	9.5	EDIC
8.2	8.1	EDIC
7.8	7.7	EDIC
11	11.2	EDIC
7.8	7.9	EDIC
9.4	9.4	EDIC
9.3	9.3	EDIC
7.9	8.1	EDIC
9.5	9.7	EDIC
10.5	10.7	EDIC
8	7.9	EDIC
9.1	9.1	EDIC
7.2	7.2	EDIC
7.8	7.8	EDIC
8.9	8.9	EDIC
9.2	9.2	EDIC
7.9	7.8	EDIC
8.1	8	EDIC
8.6	8.5	EDIC
8.3	8.5	EDIC
8.7	8.8	EDIC
7.4	7.4	EDIC
9.1	8.9	EDIC
10.9	10.9	EDIC
10	10.2	EDIC
9.7	9.6	EDIC
7.5	7.5	EDIC

Capillary HbA1c (%)	Venous HbA1c (%)	Study
8.9	8.9	EDIC
8.1	8.2	EDIC
8.8	9	EDIC
8.8	8.8	EDIC
7.7	7.8	EDIC
9.5	9.5	EDIC
9.8	9.7	EDIC
8.1	8.2	EDIC
8.6	8.4	EDIC
7.4	7.6	EDIC
4.2	8.1	EDIC
8.1	8.3	EDIC
8.8	8.9	EDIC
10.3	10.4	EDIC
7.2	7.3	EDIC
8.1	8.2	EDIC
9.4	9.5	EDIC
10.5	10.5	EDIC
8.8	8.9	EDIC
8.4	8.4	EDIC
8.9	8.9	EDIC
8.2	8.4	EDIC
8.5	8.3	EDIC
8.5	8.4	EDIC

Capillary HbA1c (%)	Venous HbA1c (%)	Study
6.7	6.6	GRADE
6.1	6	GRADE
7.8	7.9	GRADE
7.1	7.3	GRADE
7.6	7.7	GRADE
6.4	6.4	GRADE
7.1	7.1	GRADE
8.3	8.3	GRADE
6.2	6.2	GRADE
6.1	6.1	GRADE
6.3	6.4	GRADE
7.6	7.7	GRADE
6.8	6.8	GRADE
7.7	7.7	GRADE
6.5	6.5	GRADE
6.5	6.5	GRADE
7.2	6.9	GRADE
6.4	6.7	GRADE
10.8	10.7	GRADE
6.4	6.5	GRADE
6.3	6.5	GRADE
7.2	7.3	GRADE
6.8	6.9	GRADE
6.8	6.7	GRADE
6.2	6.2	GRADE
8.5	8.6	GRADE
6.4	6.4	GRADE
6.3	6.3	GRADE
6.2	6.3	GRADE
5.9	5.8	GRADE
6.9	6.9	GRADE
5.3	5.4	GRADE
6.6	6.7	GRADE
10.1	10.1	GRADE

Capillary HbA1c (%)	Venous HbA1c (%)	Study
6.4	6.4	GRADE
7.3	7.4	GRADE
5.5	5.6	GRADE
6.5	6.4	GRADE
6.9	6.8	GRADE
6.6	6.7	GRADE
6.7	6.6	GRADE
6.8	6.6	GRADE
7.1	7.2	GRADE
7.1	7.1	GRADE
7.4	7.4	GRADE
6.8	6.9	GRADE
6.2	6.3	GRADE
6.5	6.6	GRADE
6.6	6.5	GRADE
8.4	8.2	GRADE
11.9	11.9	GRADE
7.4	7.5	GRADE
10.1	9.9	GRADE
6.5	6.5	GRADE
6.6	6.6	GRADE
6.9	7	GRADE
7.7	7.7	GRADE
6.4	6.4	GRADE
7.7	7.7	GRADE
6.8	6.9	GRADE
7	7	GRADE
6.1	6.2	GRADE
9	9	GRADE
7.8	7.7	GRADE
