# Supplementary figures and images for: Comparison of central laboratory HbA1c measurements obtained from a capillary collection versus a standard venous whole blood collection in the GRADE and EDIC studies
Source: PLoS One. 2021 Nov 15;16(11):e0257154. doi: 10.1371/journal.pone.0257154 (PMC8592405; doi:10.1371/journal.pone.0257154)

S1 Fig. Map of Clinical Centers

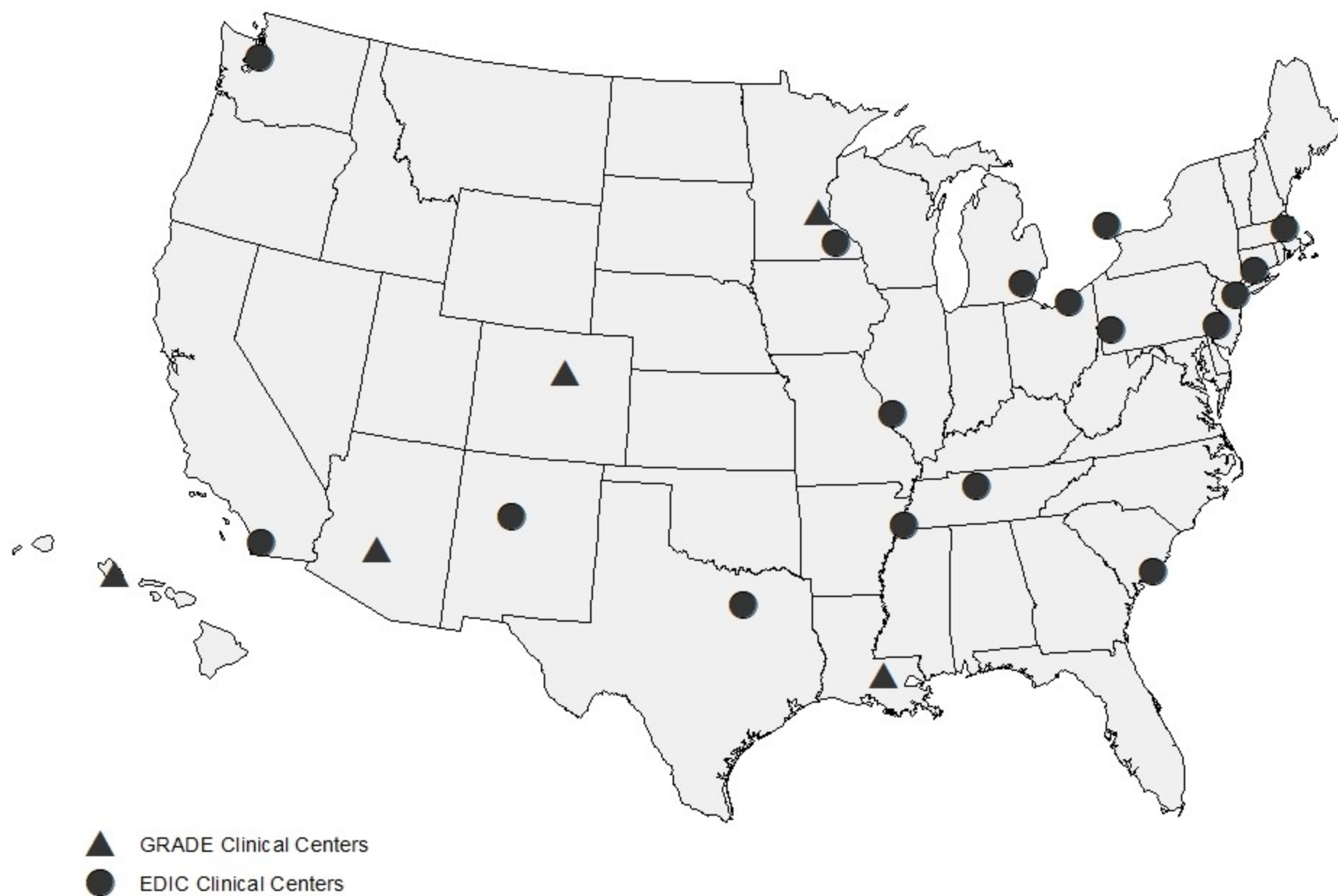

Supplement: S1 Fig — (PDF) [file pone.0257154.s002.pdf]
