# Supplementary material for: Comparison of central laboratory HbA1c measurements obtained from a capillary collection versus a standard venous whole blood collection in the GRADE and EDIC studies
Source: PLoS One. 2021 Nov 15;16(11):e0257154. doi: 10.1371/journal.pone.0257154 (PMC8592405; doi:10.1371/journal.pone.0257154)
Supplement: S2 Fig — (PDF) [file pone.0257154.s003.pdf]

S2 Fig. Participant Instructions for Capillary Blood Collection

> *Please match all contents of your kit with the items shown below.*
> ***Do not start** until you have identified all items and reviewed all steps.*

Contents of this kit for collection of HbA1c sample:

Plastic ziplock bag for returning collected sample

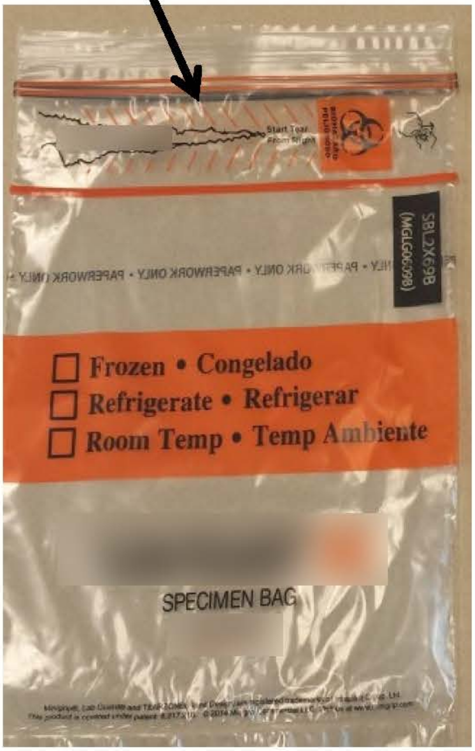

Gauze (1.5” x 1.5”)

Absorbent square (3” x 3”)

Blue-top collection vial

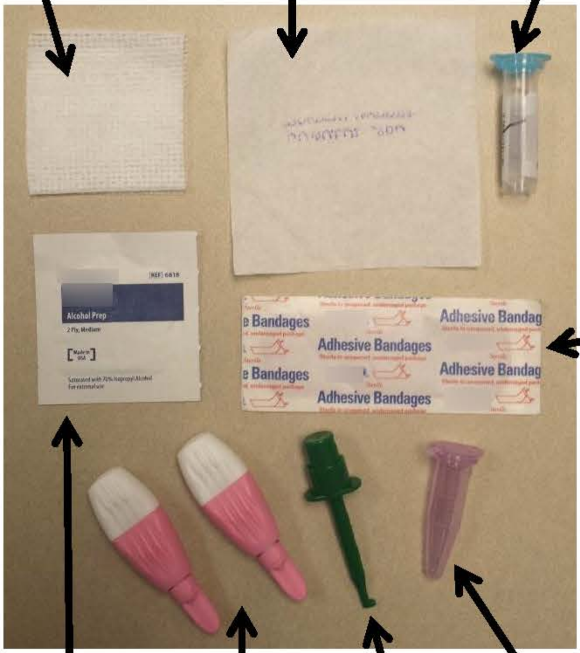

bandage

Alcohol wipe

Pink lancets (2)

Green tube holder

Container with 2 small clear plastic tubes inside

Gel pack

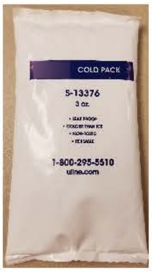

Pre-addressed, postage-paid mailer

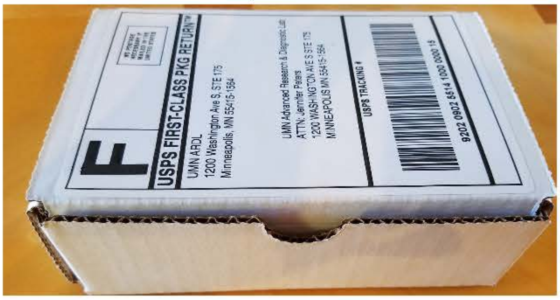

> *Unless a specific color of the item is noted, some kit contents may vary in color or packaging from that pictured.*

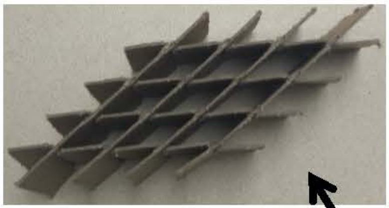

Cardboard stand for collection vial

Please read all directions before starting the sample collection. Make sure you understand the directions.

1. Place the gel pack in your freezer and allow to freeze solid. It is best to do this the day before you plan to ship your sample.

2.

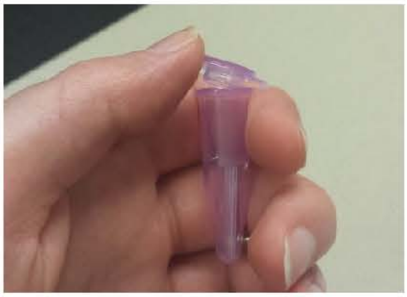

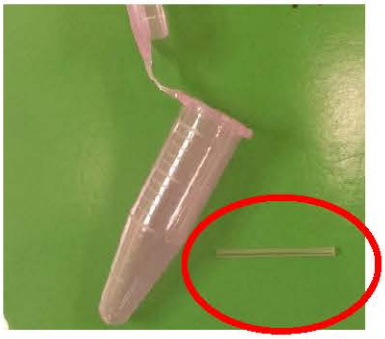

Pour out the small clear plastic tubes from the container onto a countertop or area with a color background to make the tubes easy to see. You will use **one** tube. There is an extra tube in the kit to use, if needed.

3.

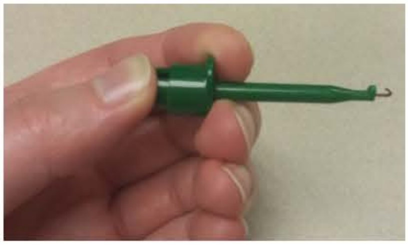

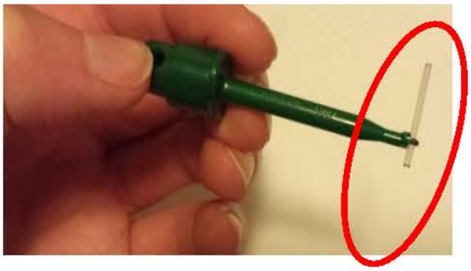

Press end of green tube holder to extend hook, place tube in hook, then release end of tube holder to secure the tube.

4.

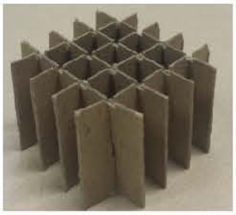

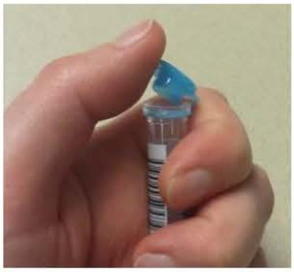

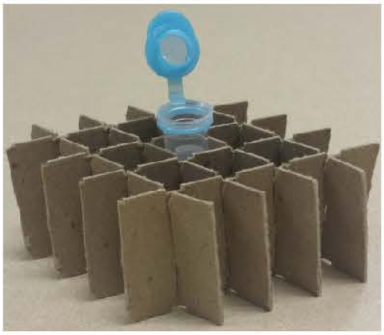

Set cardboard stand upright. Open cap on the blue-top collection vial (it is pre-labeled with a barcode sticker). There is liquid in this tube—so please use care when opening!

Place open blue-top vial in the center of the cardboard stand to hold the vial upright. This will prevent the vial from tipping over and the liquid from spilling out. ***Do not allow liquid to spill from tube.***

5. Use **one** lancet to prick your finger. An extra lancet is provided to use only if needed. If you want to, you may use your own lancet device to prick your finger instead of using the provided lancet.

To get good blood flow: place hand at your side with finger tips pointing toward the floor; shake your hand. You may also massage your hand starting at the base of the hand and moving to the fingertip; point fingers downward while massaging.

a. Clean your fingertip using alcohol wipe, and allow fingertip to dry completely.

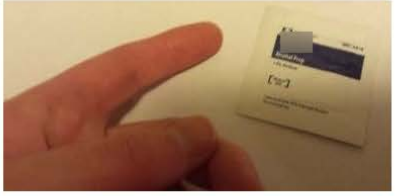

b. Twist tip off end of **one** lancet.

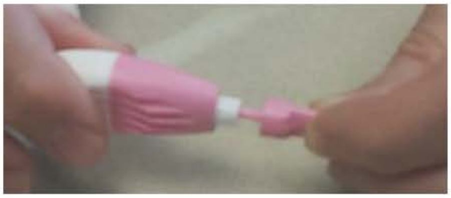

c. Press open end of lancet to fingertip until you hear a 'click.'

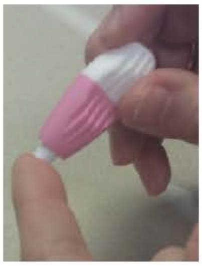

6. Using the green tube holder, touch the end of tube to blood drop on your finger. Allow tube to fill **completely** with blood. It must be filled from end-to-end. **If air bubble(s) forms in the tube, touch gauze square to end of tube to remove all blood from tube. Re-touch end of tube to blood drop on your finger to refill.

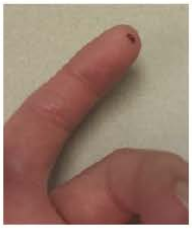

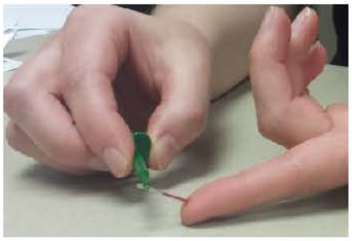

7. Press the end of tube holder to drop the tube into the collection vial.

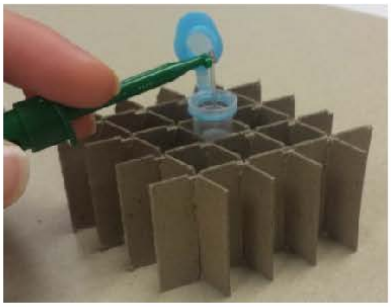

8. Apply bandage to fingertip if needed.

9. Close vial tightly. **SHAKE HARD** to remove blood completely from tube. You are done when the liquid in the vial turns slightly pink. Leave the tube in the vial.

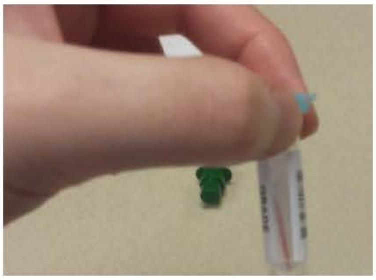

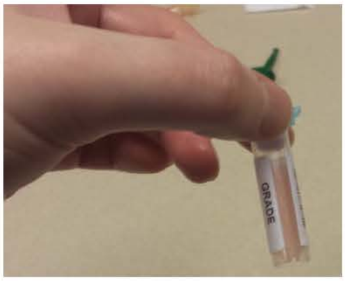

10. Complete the collection form by writing down the date you collected the blood, and any comments about the collection. GRADE staff will have already completed the other information for you.

<u>Please DO NOT write your name on this form</u>*; the laboratory will identify your sample using the barcode number on the tube.*

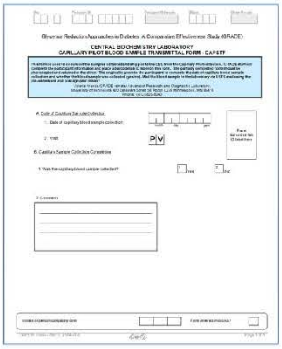

11. Package the samples for shipment:

a. Place blue-top vial, green tube holder and frozen gel pack in the plastic bag with Absorbent square. Fold the collection form and place inside plastic bag. Press out extra air and seal bag.

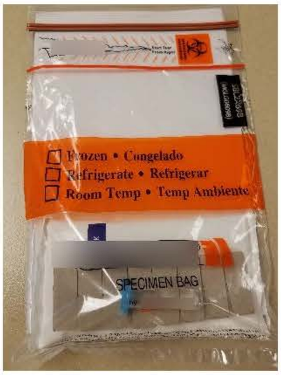

b. Fold the bag and place inside the cardboard box. To seal the box, remove liner labeled "Remove to Expose Adhesive".

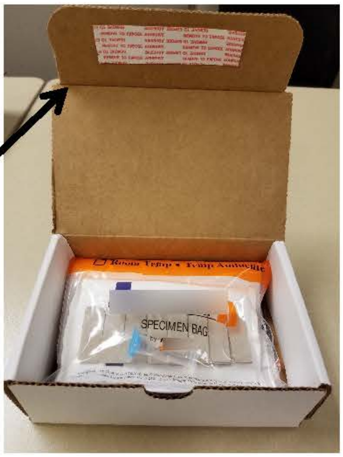

c. Press flap firmly to seal mailer.

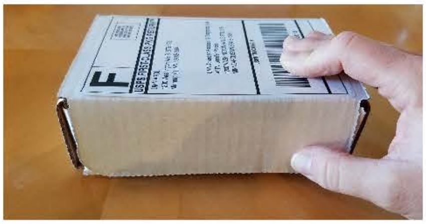

12. Place mailer in US postal mail. It may be left for a mail carrier or placed in a mail box. Mail the same day as collected or as soon as possible after collection (within 1-2 days). If it is hot outside, it is preferred that you bring the package to a USPS facility for shipping, rather than leaving in a mail box.

Do NOT write your name as the return address on the mailer;
the laboratory is not allowed to know this information.
