# Supplementary material for: Comparison of central laboratory HbA1c measurements obtained from a capillary collection versus a standard venous whole blood collection in the GRADE and EDIC studies
Source: PLoS One. 2021 Nov 15;16(11):e0257154. doi: 10.1371/journal.pone.0257154 (PMC8592405; doi:10.1371/journal.pone.0257154)
Supplement: S3 Fig — (PDF) [file pone.0257154.s004.pdf]

S3 Fig. Participant Video Instruction Postcard for Capillary Blood Collection

HbA1c Collection Kit Video

Follow the link below to watch a short video on how to use your HbA1c Collection Kit.

https://grade.bsc.gwu.edu/capkit

Thank you so much for taking the time to do the sample collection.
Your participation in GRADE is important to the study!

See reverse for important reminders mentioned in the video.

Some helpful tips for completing your collection:

- In case your shipment is lost or delayed, write the date you completed your collection here:

  ____

  (be sure to hold onto this notecard!)
- When you open the blue-capped vial, be careful not to dump or spill out the liquid
- Once the tube is filled with blood, drop it into the blue-capped vial and close the lid, then shake the vial until the liquid has turned pink.
- Fill in the date you completed the collection on the form, and write in any comments you have.
- Don’t write your name or return address anywhere on the form or mailer box.
- Mail your kit back within 2 days of collecting your sample. If it’s really hot or cold outside, drop the kit off inside a post office.
- Reach out to your GRADE team if you aren’t sure when to complete the collection, and feel free to contact them with any question you have:

  ____

  ____

  (Study Coordinator name and phone number)
