# Supplementary material for: Comparison of central laboratory HbA1c measurements obtained from a capillary collection versus a standard venous whole blood collection in the GRADE and EDIC studies
Source: PLoS One. 2021 Nov 15;16(11):e0257154. doi: 10.1371/journal.pone.0257154 (PMC8592405; doi:10.1371/journal.pone.0257154)
Supplement: S4 Fig — (PDF) [file pone.0257154.s005.pdf]

S4 Fig. GRADE Research Group (January 26, 2021)

Designations: Principal Investigator (PI); Co-Principal Investigator (Co-PI); Co-Investigator (Co-I); Study Coordinator (SC); Recruitment/Retention Coordinator (RC); Research Staff (RS)

Current Clinical Centers

Albert Einstein College of Medicine: Crandall, JP (PI); McKee, MD (Co-PI, past); Behringer-Massera , S (Co-I, past); Brown-Friday, J (SC); Xhori, E (RC, past); Ballentine-Cargill, K (RS); Duran, S (RS); Estrella, H (RS); Gonzalez de la torre, S (RS, past); Lukin, J (RS, past)

Atlanta VA Medical Center: Phillips, LS (PI); Burgess, E (Co-I); Olson, D (Co-I); Rhee, M (Co-I); Wilson, P (Co-I); Raines, TS (SC); Boers, J (SC); Costello, J (SC); Gullett, C (SC); Maher-Albertelli, M (SC); Mungara, R (SC); Savoye, L (SC); White, CA (SC); Holloway, L (SC, past); Morehead, F (SC, past); Person, S (SC, past); Sibymon, M (SC, past); Tanukonda, S (SC, past); Adams, C (RC, past); Ross, A (RC, past)

Baylor College of Medicine: Balasubramanyam, A (PI); Gaba, R (Co-I); Gonzalez, E (SC); Montes, G (RC); Wright, C (RS, past)

Baylor Research Institute: Hollander, P (PI); Roe, E (Co-I, past); Jackson, A (SC); Smiley, A (SC); Burt, P (SC, past); Estrada, L (RS); Chionh, K (RS, past)

Case Western Reserve University/Cleveland VA/MetroHealth Medical Center: Ismail-Beigi, F (PI); Falck-Ytter, C (Co-PI); Sayyed Kassem, L (Co-PI); Sood, A (Co-PI, past); Tiktin, M (Co-I, SC); Kulow, T (SC); Newman, C (SC); Stancil, KA (SC); Cramer, B (SC, past); Iacoboni, J (SC, past); Kononets, MV (SC, past); Sanders, C (SC, past); Tucker, L (SC, past); Werner, A (SC, past);Maxwell, A (RS); McPhee, G (RS); Patel, C (RS); Colosimo, L (RS, past); Krol, A (RS, past)

Columbia University Medical Center: Goland, R (PI); Pring, J (SC); Alfano, L (SC); Kringas, P (SC, past); Hausheer, C (RC, past); Tejada, J (RC past); Gumpel, K (RS, past); Kirpitch, A (RS, past); Schneier, H (RS, past)

Duke University Medical Center: Green, JB (PI); AbouAssi, H (Co-I); Chatterjee, R (Co-I); Feinglos, MN (Co-I, past); English Jones, J (SC, RC); Khan, SA (SC, RC); Kimpel, JB (SC, past); Zimmer, RP (SC, past); Furst, M (RC, past); Satterwhite, BM (RS); Evans Kreider, K (RS, past); Thacker, CR (RS)

Indiana University: Mariash, CN (PI); Mather, KJ (PI, past); Lteif, A (Co-I, past); Mullen, M (SC); Hamilton, T (SC, past); Patel, N (SC, past); Riera, G (RC); Jackson, M (RC, past); Pirics, V (RC, past); Aguillar, D (RS, past); Howard, D (RS, past); Hurt, S (RS, past)

International Diabetes Center: Bergenstal, R (PI); Carlson, A (Co-I); Martens, T (Co-I); Johnson, M (SC); Hill, R (SC); Hyatt, J (SC); Jensen, C (SC); Madden, M (SC); Martin, D (SC); Willis, H (SC); Konerza, W (RS); Yang, S (RS); Kleeberger, K (RS, past); Passi, R (RS, past)

Kaiser Permanente Northwest: Fortmann, S (PI); Herson, M (Co-I); Mularski, K (Co-I); Glauber, H (Co-I, past); Prihoda, J (Co-I, past); Ash, B (SC); Carlson, C (SC); Ramey, PA (SC); Schield, E (SC); Torgrimson-Ojerio, B (SC); Arnold, K (SC, past); Kauffman, B (SC, past); Panos, E (SC, past); Sahnow, S (RC); Bays, K (RS); Berame, K (RS); Cook, J (RS); Ghioni, D (RS); Gluth, J (RS); Schell, K (RS); Criscola, J (RS, past); Friason, C (RS, past); Jones, S (RS, past); Nazarov, S (RS, past)

Kaiser Permanente of Georgia: Barzilay, J (PI); Rassouli, N (Co-PI); Puttnam, R (Co-I); Ojoawo, B (SC); Stokes, K (SC); Nelson, R (RC); Curtis, M (SC, past); Hollis, B (SC, past); Sanders-Jones, C (SC, past); Nelson, R (RC); El-Haqq, Z (RS, past); Kolli, A (RS, past); Tran, T (RS, past)

Massachusetts General Hospital: Wexler, D (PI); Larkin, M (Co-I); Meigs, J (Co-I); Chambers, B (SC, past); Dushkin, A (SC, past); Rocchio, G (SC, past); Yepes, M (SC, past); Steiner, B (RC); Dulin, H (RC, past); Cayford, M (RS); Chu, K (RS); DeManbey, A (RS); Hillard, M (RS); Martin, K (RS); Thangthaeng, N (RS); Gurry, L (RS, past); Kochis, R (RS, past); Raymond, E (RS, past); Ripley, V (RS, past); Stevens, C (RS, past)

MedStar Health Research Institute/ MedStar Baltimore: Park, J (PI); Aroda, V (PI, past); Ghazi, A (Co-PI); Ressing, Ann (Co-I); Loveland, A (SC); Hamm, M (SC); Hurtado, M (SC); Kuhn, A (SC); Mofor, F (SC)

Miami VA Healthcare System/ University of Miami: Garg, R (PI), Lagari, V (PI); Florez, HJ (PI, past); Valencia, WM (PI, past); Marks, J (Co-PI, past); Casula, S (Co-I); Oropesa-Gonzalez, L (SC); Hue, L (SC); Riccio Veliz, AK (SC); Nieto-Martinez, R (SC, past); Gutt, M (RC)

Oregon Health & Science University: Ahmann, A (PI); Aby-Daniel, D (Co-I); Joarder, F (Co-I); Morimoto, V (Co-I); Sprague, C (Co-I); Yamashita, D (Co-I); Cady, N (SC); Rivera-Eschright, N (SC); Kirchhoff, P (SC, past); Morales Gomez, B (RC); Adducci, J (RC, past); Goncharova, A (RC, past)

Pacific Health Research & Education Institute/VA Pacific Islands: Hox, SH (PI); Petrovitch, H (Co-PI); Matwichyna, M (SC); Jenkins, V (SC, past); Bermudez, NO (RS); Broadwater, L (RS); Ishii, RR (RS)

Pennington Biomedical Research Center: Hsia, DS (PI); Cefalu, WT (PI, past); Greenway, FL (Co-I); Waguespack, C (Co-I); King, E (SC); Haynes, N (SC, past); Thomassie, A (SC, past); Bourgeois, B (RC, past); Hazlett, C (RS)
San Diego VA Medical Center: Mudaliar, S (PI); Henry, R (PI, past); Boeder, S (Co-I, past); Pettus, J (Co-I, past); Diaz, E (SC); Garcia-Acosta, D (SC); Maggs, S (SC); DeLue, C (SC, past); Castro, E (RC, past); Hernandez, S (RC, past)
Southwestern American Indian Center: Krakoff, J (PI); Curtis, JM (Co-I); Killean, T (SC); Khalid, M (SC); Joshevama, E (RC, past); Diaz, E (RS); Martin, D (RS); Tsingine, K (RS); Karshner, T (RS, past)
St. Luke's-Roosevelt Hospital: Albu, J (Co-PI); Pi-Sunyer, FX (Co-PI, past); Frances, S (SC); Maggio, C (SC, past); Ellis, E (RC); Bastawrose, J (RC, past); Gong, X (RS)
SUNY Downstate Medical Center/New York Hospital-Queens: Banerji, MA (PI); August, P (Co-I); Lee, M (Co-I); Lorber, D (Co-I); Brown, NM (SC, RC); Josephson, DH (SC); Thomas, LL (SC, RC); Tsovian, M (SC, RC); Cherian, A (SC, RC, past); Jacobson, MH (RS); Mishko, MM (RS)
The University of North Carolina Diabetes Care Center: Kirkman, MS (PI); Buse, JB (Co-I); Dostou, J (Co-I); Young, L (Co-I); Bergamo, K (Co-I, past); Goley, A (Co-I, past); Kerr, J (Co-I, past); Largay, JF (Co-I, past); Guarda, S (SC); Cuffee, J (SC, past); Culmer, D (SC, past); Fraser, R (RC); Almeida, H (RC, past); Coffer, S (RC, past); Debnam, E (RC, past); Kiker, L (RC, past); Morton, S (RC, past); Josey, K (RS); Fuller, G (RS, past)
University of Alabama Birmingham: Garvey, WT (PI); Cherrington, A (Co-PI); Golson, D (SC); Robertson, MC (SC); Griffith, O (SC, past); Agne, A (RC); McCullars, S (RC)
University of Cincinnati/ Cincinnati VA Medical Center: Cohen, RM (PI); Craig, J (SC); Rogge, MC (SC); Burton, K (SC, past); Kersey, K (SC, RC, past); Wilson, C (SC, past); Lipp, S (RC, past); Vonder Meulen, MB (RC, past)
University of Colorado-Denver/VA: Rasouli, N (PI); Baker, C (Co-I); Schroeder, E (Co-I, past); Underkofler, C (SC); Lorch, R (SC); Douglass, S (SC, past); Steiner, S (SC, past)
University of Iowa: Sivitz, W (PI); Cline, E (SC); Knosp, L (SC); McConnell, J (SC, past); Lowe, T (RC)
University of Michigan: Herman, WH (PI); Pop-Busui, R (Co-PI); Tan, MH (Co-I); Martin, C (SC); Waltje, A (SC, RC); Katona, A (SC); Goodhall, L (SC, past); Eggleston, R (RC, past); Kuo, S (RS); Whitley, K (RS); Bule, S (RS, past); Kessler, N (RS, past); LaSalle, E (RS, past)
University of Minnesota: Seaquist, ER (PI); Bantle, A (Co-I); Harindhanavudhi, T (Co-I); Kumar, A (Co-I); Redmon, B (Co-I); Bantle, J (Co-I, past); Coe, M (SC); Mech, M (SC); Taddese, A (RC); Lesne, L (RS); Smith, S (RS)
University of Nebraska Medical Center/Omaha VA: Desouza, C (PI); Kuechenmeister, L (Co-I); Shivaswamy, V (Co-I); Morales, AL (SC); Rodriguez, MG (SC); Seipel, K (SC); Alfred, A (SC, past); Eggert, J (RS); Lord, G (RS); Taylor, W (RS, past); Tillson, R (RS, past)
University of New Mexico: Schade, DS (PI); Adolphe, A (Co-PI); Burge, M (Co-PI, past); Duran-Valdez, E (SC); Martinez, J (RC, past); Kunkel, S (RS); Ali Jamaleddin Ahmad, F (RS, past); Hernandez McGinnis, D (RS, past); Pucchetti, B (RS, past); Scripsick, E (RS, past); Zamorano, A (RS, past)
UT Health San Antonio: DeFronzo, RA (PI); Cersosimo, E (Co-PI); Abdul-Ghani, M (Co-I); Triplitt, C (Co-I); Mullen, M (SC); Garza, RI (SC, past); Verastiqui, H (SC, past); Wright, K (RC, past); Puckett, C (RS)
University of Texas-Southwestern Medical Center: Raskin, P (PI); Rhee, C (Co-I, past); Abraham, S (SC); Jordan, LF (SC); Sao, S (SC); Morton, L (SC, past); Smith, O (SC, past); Osornio Walker, L (RC, past); Schnurr-Breen, L (RC, past); Ayala, R (RS); Kraymer, RB (RS); Sturgess, D (RS, past)
VA Puget Sound Healthcare System/University of Washington: Utzschneider, KM (PI); Kahn, SE (Co-PI); Alarcon-Casas Wright, L (Co-I); Boyko, EJ (Co-I); Tsai, EC (Co-I); Trence, DL (Co-I, past); Fattaleh, BN (SC); Montgomery, BK (SC, past); Atkinson, KM (RS); Kozedub, A (RS); Concepcion, T (RS, past); Moak, C (RS, past); Prikhodko, N (RS, past); Rhothisen, S (RS, past)
Vanderbilt University: Elasy, TA (PI); Martin, S (SC); Shackelford, L (RC, RS); Goidel, R (RS); Hinkle, N (RS); Lovell, C (RS); Myers, J (RS); Lipps Hogan, J (RS, past)
Washington University: McGill, JB (PI); Salam, M (Co-I); Schweiger, T (SC, RC); Kissel, S (SC, RC, past); Recklein, C (SC, past); Clifton, MJ (RS)
Yale University/Fair Haven Community Health Center/West Haven VA Medical Center: Tamborlane, W (PI); Camp, A (Co-I); Gulanski, B (Co-I); Inzucchi, SE (Co-I); Pham, K (Co-I); Alguard, M (SC, RC); Gatcomb, P (SC); Lessard, K (SC); Perez, M (SC); Iannone, L (RC); Magenheimer, E (RC); Montoza, A (RC)

Study Units

NIH/NIDDK (Sponsor): Cefalu, W (Director); Burch, HB (Project Scientist); Bremer, A (Program Scientist, past); Linder, B (Program Official); Fradkin, J (Director, past)

Chairman's Office, Massachusetts General Hospital, Harvard Medical School: Nathan, DM (Study Chair, Study Co-PI)

Executive Committee: Nathan, DM (Study Chair, Study Co-PI); Lachin, JM (U01 Contact PI, Study Co-PI); Buse, JB (Co-I); Kahn, SE (Co-I); Krause-Steinrauf, H (Co-I, Project Director); Larkin, M (Co-I); Tiktin, M (Co-I, SC); Wexler, D (PI); Burch, HB (Program Scientist); Linder, B (Program Official); Bremer, A (Program Scientist, past)

Coordinating Center, The George Washington University Biostatistics Center: Lachin, JM (U01 Contact PI, Study Co-PI); Krause-Steinrauf, H (Co-I, Project Director); Younes, N (Co-I); Backman, M (RS); Bebu, I (RS); Buys, CJ (RS); Fagan Murphy, A (RS); Gao, Y (RS); Gramzinski, MR (RS); Hall, SD (RS); Legowski, E (RS); Suratt, C (RS); Arey, A (RS, past); Bethepu, J (RS, past); Lund, C (RS, past); Mangat Dhaliwal, P (RS, past); McGee, P (RS, past); Mesimer, E (RS, past); Ngo, L (RS, past)

Central Biochemical Laboratory, University of Minnesota Advanced Research & Diagnostic Laboratory: Steffes, M (PI); Seegmiller, J (Co-I); Saenger, A (Co-I, past); Arends, V (SC); Gabrielson, D (SC, past)

Drug Distribution Center, VA Cooperative Studies Program Clinical Research Pharmacy Coordinating Center: Conner, T (PI); Warren S (PI, past); Day, J (RS); Scrymgeour, A (RS)

ECG Reading Center, EPICARE, Wake Forest University: Pokharel, Y (PI); Soliman, EZ (PI, past); Zhang, ZM (Co-I, past); Campbell, C (SC); Keasler, L (SC); Hensley, S (SC, past); Hu, J (SC, past); Li, Y (RS)

Economic Evaluation and Assessment Center:

University of Michigan: Herman, W (PI); Martin, C (SC); Waltje, A (SC, RC); Kuo, S (RS); Mihalcea, R (RS); Min, DJ (RS); Perez-Rosas, V (RS); Prosser, L (RS); Resnicow, K (RS); Ye, W (RS)

Centers for Disease Control and Prevention: Shao, H (RS); Zhang, P (RS)

Neurocognitive Coordinating Center, Columbia University Medical Center: Luchsinger, J (PI); Sanchez, D (SC); Assuras, S (RS)

QWB Reading Center, University of California San Diego Health Services Research Center: Groessl, E (PI); Sakha, F (SC); Hillery, N (RS) Chong, H (SC, past)

Collaborators

Cardiovascular Adjudication Advisor: Everett, B (Brigham and Women's Hospital)

Collaborating Investigators (Recruitment Site): Abdouch, I (University of Nebraska Medical Center/Omaha VA); Brantley, P (Pennington Biomedical Research Center (LSU)); Broyles, FE (SIBCR – VA Puget Sound); Canaris, G (University of Nebraska Medical Center/Omaha VA); Copeland, P (Massachusetts General Hospital); Craine, JJ (SIBCR – VA Puget Sound); Fein, WL (SIBCR – VA Puget Sound); Lee, MS (SUNY Downstate Medical Center); Meiners, R (Pennington Biomedical Research Center (LSU)); Meiners, V (Pennington Biomedical Research Center (LSU)); O'Neal, H (Pennington Biomedical Research Center (LSU)); Park, JE (SIBCR – VA Puget Sound); Sledge, Jr., E (Pennington Biomedical Research Center (LSU)); Steppel-Resnick, J (Massachusetts General Hospital); Turchin, A (Massachusetts General Hospital)

Beta Cell Ancillary Study: Brooks-Worrell, B (University of Washington); Hampe, CS (University of Washington); Newgard, CB (Duke University); Palmer, JP (University of Washington); Shojaie, A (University of Washington)

Continuous Glucose Monitoring Sub-study: Higgins, J (Massachusetts General Hospital; Harvard Medical School)

Emotional Distress Sub-study: Fischer, L (University of California, San Francisco); Golden, S (Johns Hopkins University); Gonzalez, J (Albert Einstein College of Medicine); Naik, A (Baylor College of Medicine); Walker, E (Albert Einstein College of Medicine)

National Diabetes Education Program (NDEP) Sub-study: Doner Lotenberg, L (Hager Sharp); Gallivan, JM (National Institutes of Health); Lim, J (Hager Sharp); Tuncer, DM (National Institutes of Health)

Recruitment Ancillary Study: Behringer-Massera, S (The Mount Sinai Hospital, Beth Israel Medical Center)
