# Supplementary material for: Comparison of central laboratory HbA1c measurements obtained from a capillary collection versus a standard venous whole blood collection in the GRADE and EDIC studies
Source: PLoS One. 2021 Nov 15;16(11):e0257154. doi: 10.1371/journal.pone.0257154 (PMC8592405; doi:10.1371/journal.pone.0257154)
Supplement: S5 Fig — (PDF) [file pone.0257154.s006.pdf]

S5 Fig. DCCT/EDIC Research Group (January 1, 2021)

Study Chairpersons – D.M. Nathan (chair), B. Zinman (vice-chair); *Past*: O. Crofford; *Deceased*: S. Genuth

Editor, EDIC Publications – D.M. Nathan

Clinical Centers

Case Western Reserve University – *Current*: R. Gubitosi-Klug, L. Mayer, J. Wood, D. Miller, A. Nayate, M. Novak, S. Pendegast, L. Singerman, D. Weiss, H. Zegarra; *Past*: E. Brown, P. Crawford, M. Palmert, P. Pugsley, J. Quin, S. Smith-Brewer; *Deceased*: W. Dahms, S. Genuth, J. McConnell

Weill Cornell Medical College – *Current*: N.S. Gregory, R. Hanna, R. Chan, S. Kiss, A. Orlin, M. Rubin; *Past*: S. Barron, B. Bosco, D. Brillon, S. Chang, A. Dwoskin, M. Heinemann, L. Jovanovic, M.E. Lackaye, T. Lee, B. Levy, V. Reppucci, M. Richardson; *Deceased*: R. Campbell

Henry Ford Health System – *Current*: A. Bhan, J.K. Jones, D. Kruger, P.A. Edwards, H. Remtema; *Past*: E. Angus, A. Galprin, M. McLellan, A. Thomas; *Deceased*: J.D. Carey, F. Whitehouse

International Diabetes Center – *Current*: R. Bergenstal, S. Dunnigan, M. Johnson, A. Carlson, ; *Past*: R. Birk, P. Callahan, G. Castle, R. Cuddihy, M. Franz, D. Freking, L. Gill, J. Gott, K. Gunyou, P. Hollander, D. Kendall, J. Laechelt, S. List, W. Mestrezat, J. Nelson, B. Olson, N. Rude, M. Spencer, L. Thomas; *Deceased*: D. Etzwiler, K. Morgan

Joslin Diabetes Center – *Current*: L.P. Aiello, E. Golden, P. Arrigg, R. Beaser, L. Bestourous, J. Cavallerano, R. Cavicchi, O. Ganda, O. Hamdy, T. Murtha, D. Schlossman, S. Shah, G. Sharuk, P. Silva, P. Silver, M. Stockman, J. Sun, E. Weimann; *Past*: V. Asuquo, A. Jacobson, R. Kirby, L. Rand, J. Rosenzwieg, H. Wolpert

Massachusetts General Hospital – *Current*: D.M. Nathan, M.E. Larkin, M. Cayford, A. deManbey, L . Gurry, J. Heier, A. Joseph, F. Leandre, K. Martin, C. Shah, C. Stevens, N. Thangthaeng; *Past*: E. Anderson, H. Bode, S. Brink, M. Christofi, C. Cornish, D. Cros, S. Crowell, L. Delahanty, K. Folino, S. Fritz, C. Gauthier-Kelly, J. Godine, C. Haggan, K. Hansen, P. Lou, J. Lynch, C. McKitrick, D. Moore, D. Norman, M. Ong, E. Ryan, C. Taylor, D. Zimbler

Mayo Clinic – *Current*: A. Vella, A. Zipse, A. Barkmeier; *Past*: B. French, M. Haymond, J. Mortenson, J. Pach, R. Rizza, L. Schmidt, W.F. Schwenk, F.J. Service, R. Woodwick, G. Ziegler; *Deceased*: R. Colligan, A. Lucas, B. Zimmerman

Medical University of South Carolina – *Current*: H. Karanchi, L. Spillers, J. Fernandes, K. Hermayer, S. Kwon, K. Lee, M. Lopes-Virella, T. Lyons, M. Nutaitis; *Past*: A. Blevins, M. Bracey, S. Caulder, J. Colwell, S. Elsing, A. Farr, D. Lee, P. Lindsey, L. Luttrell, R. Mayfield, J. Parker, N. Patel, C. Pittman, J. Selby, J. Soule, M. Szpiech, T. Thompson, D. Wood, S. Yacoub-Wasef

Northwestern University – *Current*: A. Wallia, M. Hartmuller, S. Ajroud-Driss, P. Astelford, A. Degillio, M. Gill, L. Jampol, C. Johnson, L. Kaminski, N. Leloudes, A. Lyon, R. Mirza, D. Ryan, E. Simjanoski, Z. Strugula; *Past*: D. Adelman, S. Colson, M. Molitch, B. Schaefer

University of California, San Diego – *Current*: S. Mudaliar, G. Lorenzi, O. Kolterman, M. Goldbaum; *Past*: T. Clark, M. Giotta, I. Grant, K. Jones, R. Lyon, M. Prince, R. Reed, M. Swenson; *Deceased*: G. Friedenberg

University of Iowa – *Current*: W.I. Sivitz, B. Vittetoe, J. Kramer; *Past*: M. Bayless, C. Fountain, R. Hoffman, J. MacIndoe, N. Olson, H. Schrott, L. Snetselaar, T. Weingeist, R. Zeitler

University of Maryland – *Current*: R. Miller, S. Johnsonbaugh; *Past*: M. Carney, D. Counts, T. Donner, J. Gordon, M. Hebdon, R. Hemady, B. Jones, A. Kowarski, R. Liss, S. Mendley, D. Ostrowski, M. Patronas, P. Salemi, S. Steidl

University of Michigan – *Current*: W.H. Herman, R. Pop-Busui, C.L. Martin, P. Lee, J. W. Albers, E.L. Feldman; *Past*: N. Burkhart, D.A. Greene, T. Sandford, M.J. Stevens; *Deceased*: J. Floyd

University of Minnesota – *Current*: J. Bantle, M. Rhodes, D. Koozekanani, S. Montezuma, J. Terry; *Past*: N. Flaherty, F. Goetz, C. Kwong, L. McKenzie, M. Mech, J. Olson, B. Rogness, T. Strand, R. Warhol, N. Wimmergren

University of Missouri – *Current*: D. Goldstein, D. Hainsworth, S. Hitt; *Deceased*: J. Giangiacomo

University of New Mexico – *Current*: D.S. Schade, J.L. Canady, R.B. Avery, M.R. Burge, J.E. Chapin, A. Das, L.H. Ketai; *Past*: D. Hornbeck, C. Johannes, J. Rich, M.L Schluter

University of Pennsylvania – *Current*: M. Schutta, P.A. Bourne, A. Brucker; *Past*: S. Braunstein, B.J. Maschak-Carey, S. Schwartz; *Deceased*: L. Baker

University of Pittsburgh – *Current*: T. Orchard, L. Cimino, D. Rubinstein; *Past*: D. Becker, B. Doft, D. Finegold, K. Kelly, L. Lobes, N. Silvers, T. Songer, D. Steinberg, L. Steranchak, J.Wesche; *Deceased*: A. Drash

University of South Florida – *Current*: J.I. Malone, A. Morrison, M.L. Bernal, P.R. Pavan; *Past*: L. Babbione, T.J. DeClue, N. Grove, D. McMillan, H. Solc, E.A. Tanaka, J. Vaccaro-Kish

University of Tennessee – *Current*: S. Dagogo-Jack, C. Wigley, S. Huddleston, A. Patel; *Past*: M. Bryer-Ash, E. Chaum, A. Iannacone, H. Lambeth, D. Meyer, S. Moser, M.B. Murphy, H. Ricks, S. Schussler, S. Yoser; *Deceased*: A. Kitabchi

University of Texas – *Current*: P. Raskin, S. Strowig, YG. He, E. Mendelson, RL. Ufret-Vincenty; *Past*: M. Basco; *Deceased*: S. Cercone

University of Toronto – *Current*: B.A. Perkins, B. Zinman, A. Barnie, N. Bakshi, M. Brent, R. Devenyi, K. Koushan, M. Mandelcorn, F. Perdikaris, L. Tuason; *Past*: D. Daneman, R. Ehrlich, S. Ferguson, A. Gordon, K. Perlman, S. Rogers

University of Washington – *Current*: I. Hirsch, R. Fahlstrom, L. Van Ottingham, I.H. de Boer, L. Olmos de Koo; *Past*: S. Catton, J. Ginsberg, J. Kinyoun, J. Palmer

University of Western Ontario – *Current*: C. McDonald, M. Driscoll, J. Bylsma, T. Sheidow; *Past*: W. Brown, C. Canny, P. Colby, S. Debrabandere, J. Dupre, J. Harth, I. Hramiak, M. Jenner, J. Mahon, D. Nicolle, N.W. Rodger, T. Smith

Vanderbilt University – *Current*: M. May, J. Lipps Hagan, T. Adkins, A. Agarwal, C. Lovell; *Past*: S. Feman, R. Lorenz, R. Ramker; *Deceased*: L. Survant

Washington University, St. Louis – *Current*: N.H. White, L. Levandoski; *Deceased*: I. Boniuk, J. Santiago

Yale University – *Current*: W. Tamborlane, P. Gatcomb, K. Stoessel; *Past*: J. Ahern, K. Fong, P. Ossorio, P. Ramos

Albert Einstein – *Past*: J. Brown-Friday, J. Crandall, H. Engel, S. Engel, H. Martinez, M. Phillips, M. Reid, H. Shamoon, J. Sheindlin

Clinical Coordinating Center

Case Western Reserve University – *Current*: R. Gubitosi-Klug, L. Mayer, C. Beck, K. Farrell, P. Gaston; *Past*: S. Genuth, M. Palmert, J. Quin, R. Trail; *Deceased*: W. Dahms

Data Coordinating Center

George Washington University, The Biostatistics Center – J. Lachin, I. Bebu, B. Braffett, J. Backlund, L. Diminick, L. El ghormli, X. Gao, D. Kenny, K. Klumpp, M. Lin, V. Trapani; *Past*: K. Anderson, K. Chan, P. Cleary, A. Determan, L. Dews, W. Hsu, P. McGee, H. Pan,B. Petty, D. Rosenberg, B. Rutledge, W. Sun, S. Villavicencio, N. Younes; *Deceased*: C. Williams

National Institute of Diabetes and Digestive and Kidney Disease

National Institute of Diabetes and Digestive and Kidney Disease Program Office – E. Leschek; *Past*: C. Cowie, C. Siebert

EDIC Core Central Units

Central Biochemistry Laboratory (University of Minnesota) – M. Steffes, A. Karger, J. Seegmiller, V. Arends; *Past*: J. Bucksa, B. Chavers, A. Killeen, M. Nowicki, A. Saenger

Central ECG Reading Unit (Wake Forest School of Medicine) – Y. Pokharel, M. Barr, C. Campbell, S. Hensley, J. Hu, L. Keasler, Y. Li, T. Taylor, Z.M. Zhang; *Past*: R. Prineas, E.Z. Soliman

Central Ophthalmologic Reading Unit (University of Wisconsin) – B. Blodi, R. Danis, D. Lawrence, H. Wabers; *Past*: M. Burger, M. Davis, J. Dingledine, V. Gama, S. Gangaputra, L. Hubbard, S. Neill, R. Sussman

Central Neuropsychological Reading Unit (NYU Winthrop Hospital, University of Pittsburgh) – A. Jacobson, C. Ryan, D. Saporito; *Past*: B. Burzuk, E. Cupelli, M. Geckle, D. Sandstrom, F. Thoma, T. Williams, T. Woodfill
